# Imaging of the Equine Abdomen Using Point of Care Ultrasound (POCUS)—A Resource for the Equine Practitioner

**DOI:** 10.3390/ani16121770

**Published:** 2026-06-08

**Authors:** Francesca Freccero, Barbara Padalino, Ann Carstens, Sharanne L. Raidal

**Affiliations:** 1 Department of Veterinary Medical Sciences, University of Bologna, 40126 Bologna, Italy; francesca.freccero2@unibo.it; 2 Faculty of Science and Engineering, Southern Cross University, Lismore 2480, Australia; barbara.padalino@scu.edu.au; 3 Department of Agricultural and Food Sciences, University of Bologna, 40126 Bologna, Italy; 4 School of Agricultural, Environmental and Veterinary Sciences, Charles Sturt University, Wagga Wagga 2650, Australia; acarstens@csu.edu.au; 5 Melbourne Veterinary School, The University of Melbourne, Werribee 3030, Australia

**Keywords:** sonography, ultrasonography, ultrasonographic, FLASH, colic, acute abdomen, gastrointestinal motility, horse

## Abstract

Wireless ultrasound (US) probes for point of care (POC) evaluation of horses presented for veterinary evaluation of colic have become widely available in recent years. The availability and performance of this technology mean POCUS is readily available to the equine practitioner and the technology is well suited to patient evaluation or serial monitoring in first opinion practice. In human health care and veterinary contexts, it has been suggested that there is a need for practitioners to better appreciate the value of POCUS for assessment of gastrointestinal structure and function. This study was conducted as a narrative review of the literature relating to the use of abdominal sonography in equine clinical practice, juxtaposed with recent publications on the use of POCUS in human health care settings and extended to include the authors’ experiences of POCUS in the evaluation of horses presenting with select abdominal conditions. Common sonographic views are described and representative images are presented to facilitate practitioner use of the technique. By distillation of the available literature and review of sonographic techniques our aim is to provide a resource for equine practitioners.

## Simple Summary

Small, hand-held devices are in common use for point of care ultrasound (POCUS) examinations in human health care settings. These probes connect wirelessly (or by cable) to smartphones or tablets to provide the clinician with ultrasound images of the patient in real-time. Rather than replacing specialist imaging procedures, this approach is seen as an extension of the clinical examination of patients with abdominal and gastrointestinal disorders at the bedside or in the emergency department. The technology is readily available at a modest cost and is rapidly being implemented in equine practice, meaning that ultrasound examination of the horse’s abdomen—previously largely restricted to specialist referral practices—might now be considered part of the initial work up of horses with abdominal problems, particularly colic. In medical and veterinary settings, concern has been expressed that there is a need to support general practitioners to correctly use and interpret sonographic findings in this context. To facilitate the use of POCUS in general equine practice, this paper presents a review of common sonographic findings of the equine abdomen in health and disease, juxtaposed with advantages associated with POC sonography in the medical literature and the authors’ experience of the technique.

## 1. Introduction

Percutaneous ultrasonographic assessment of the equine abdomen has been reported using systematic [1,2,3,4,5,6,7] and focused techniques [4,8,9]. Sonographic evaluation of the colic patient facilitates recognition of changes in normal abdominal topography, including shape, size and content of viscera; gastrointestinal motility; intestinal wall thickness and layering [5]; and peritoneal fluid quantity and characteristics [6]. In horses, sonography has been utilized to characterize gastrointestinal changes associated with colic, medication, feeding and for post-operative monitoring. In most instances, a 2 to 3.5 MHz curved linear or convex transducer is required to image the peripheral equine abdomen transcutaneously [1,5,6]. Hence, despite the established value of the ultrasonographic evaluation of the abdomen in equine practice, until recently [5,9,10], equipment constraints and fiscal limitations have largely restricted the use of this technology to specialist hospital settings. Detailed knowledge of the sonographic appearance of the equine abdomen was, therefore, largely beyond the scope of the generalist equine practitioner.

Pocket-sized, hand-held, ultrasound transducers are now readily available and connect to a tablet or a smartphone device via wi-fi or cable to display sonographic images, negating the need for larger, less portable, ultrasound equipment [9]. The development and use of small bedside or point of care ultrasound (POCUS) systems has been increasingly reported in human acute care settings [11,12]. Rather than replacing specialist imaging procedures, this approach is seen as an extension of the clinical examination of patients with abdominal and gastrointestinal disorders at the bedside or in the emergency department [13,14], and is likely to be well suited to equine practice. To date, however, there is relatively little information available on the application and use of abdominal POCUS approaches in equine practice [15], and sonography of the abdomen does not feature strongly in the majority of veterinary [5,15] or medical [14] undergraduate curricula.

This study has been framed as a narrative review summarizing veterinary literature on the sonographic assessment of the equine abdomen and briefly reviewing medical experiences using POCUS for acute abdominal presentations in human patients. Narrative reviews synthesize multiple points of view and harness unique review team perspectives to shape critical analysis [16] and are particularly well suited to novel or emerging methodology [17], such as POCUS. Expert-based practical guidance and representative images from key sonographic windows, taken by the authors using hand-held wi-fi sonographic probes for the POC evaluation of horses, have been included in this review to illustrate and refine information presented. The objective of this review was to describe what is known on the topic while conducting a subjective examination and critique of available literature [18,19], and hence to provide a resource for equine practitioners to facilitate field evaluation of the adult horse presenting for the evaluation of acute abdomen.

## 2. Materials and Methods

### 2.1. Narrative Review

PubMed, Scopus, CAB Abstracts and Web of Sciences databases were interrogated until 24 February 2026 using relevant search terms (horse* OR equine) AND (abdominal ultrasound OR abdominal ultrasonography OR gastrointestinal ultrasound OR gastrointestinal ultrasonography OR POCUS). Publications were evaluated to obtain information on the clinical applications of and techniques for sonographic assessment of the equine abdomen, including equipment requirements, patient preparation and effects of common management and therapeutic interventions. Combining all databases, the search strategy yielded 701 records (Supplementary File S1). After exclusion of 250 duplicates, 451 records were screened by title and journal, with 294 records excluded as irrelevant; an additional 43 records were excluded on this basis on full-text review by one or more authors. The resultant 114 papers were reviewed by authors and a further 80 records were removed by consensus on the basis that they contributed little additional information (data saturation) on the utility of the technique, were superseded by more recent or more comprehensive studies, or were too narrow to be of general interest. The remaining 34 records (Supplementary File S1) formed the basis of this review, with authors using these publications to explore their own practices and interpretation, justifying divergent opinions based on the inclusion of additional references obtained by snowballing (i.e., references derived from selected references) and from the authors’ collections, including review of relevant textbooks, to support information presented. Select references derived from human healthcare settings were included for comparative purposes but results from small animal veterinary species were considered outside the scope of the current review. Equipment available to the equine practitioner was identified from the authors’ knowledge of veterinary equipment providers and equipment available online or through veterinary suppliers. Key information from sourced articles was collated and sorted into emergent themes shaped around our research question: *How can point of care ultrasonography (POCUS) be used in the assessment of the equine acute abdomen?*

### 2.2. Clinical Experiences

**Figure 1:**
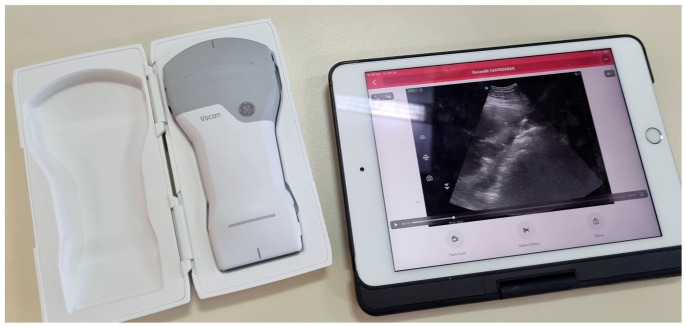
VScan Air wireless ultrasound transducer and representative image on wirelessly connected device.

Point of care sonographic assessment has been used by the authors to rapidly screen horses presenting with colic as a rapid, focused technique included in initial clinical evaluation. The technique is particularly useful where immediate decisions are required and for monitoring clinical response. Cases assessed by the authors using POCUS were reviewed and representative images collated to illustrate the expected normal sonographic appearance of frequently used acoustic windows and to demonstrate common abnormalities identified from each window. Horses were imaged using a Vscan Air CL probe (GE Healthcare, Mascot, NSW, Australia; Figure 1), a hand-held, battery-operated dual-headed ultrasonographic transducer. In addition to a linear array transducer (3–12 MHz), not used in the current study, this device has a convex 2–5 MHz transducer able to penetrate to 20–25 cm and hence is well suited to transcutaneous evaluation of the peripheral equine abdomen. The device was connected wirelessly (Bluetooth BLE 4.0) to an Android (smart phone) or iOS device (iPad) via a specific app (Vscan Air VET Ultrasound, https://apps.apple.com/lu/app/vscan-air-vet-ultrasound/id1644084883, accessed on 27 May 2026), and scans were saved as single images or image sequences (cineloops) of up to 30 s duration. Sonographic examinations were obtained as part of clinical assessment of horses presented for veterinary evaluation and hence were exempt from scrutiny by institutional animal ethics committee. The approach to abdominal ultrasound evaluation ranged between a comprehensive, methodical survey of the entire abdomen to a focused, localized assessment completed during acute presentations or repeated to monitor clinical response, at the discretion of the attending veterinarian. Sonographic imaging was not standardized but was based on case presentation, progression or context. Horses were clipped only if necessary for image acquisition or to facilitate repeat evaluation of the same acoustic window. Ethanol (70%) was sprayed or poured topically as an acoustic coupling agent. Acoustic coupling gel was applied over the transducer, which was then covered by a disposable probe cover (AtlasVet Ultrasound Probe Covers; ZebraVet Australia Pty Ltd., Braeside, Vic, Australia). Gel was, infrequently, also applied to the skin to improve contact.

## 3. Results

### 3.1. How Should Sonographic Assessment Be Performed in Horses?

**Figure 2:**
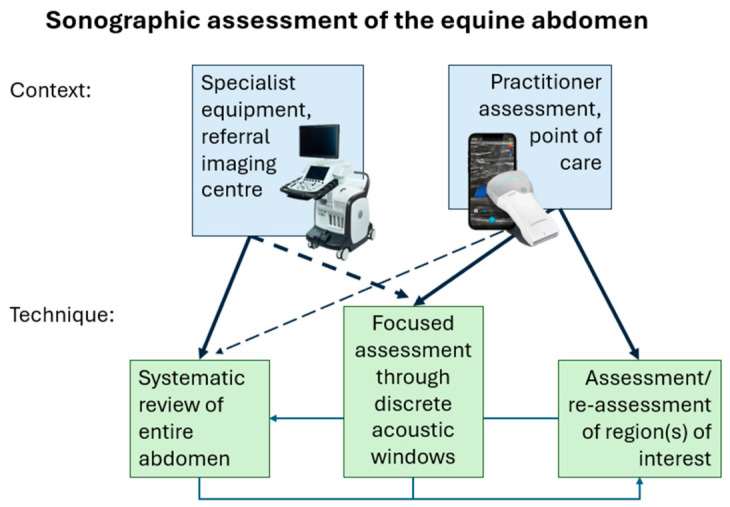
Sonographic assessment of the equine abdomen may be completed in specialist imaging centres using high-end equipment, which typically requires moving the horse to the facility, or by using point of care equipment designed for use stall-side or in ambulatory practice. Depending on the clinical condition of the patient, comprehensive and systematic review of the entire abdomen or more rapid evaluation using pre-determined acoustic windows may be appropriate. Discrete regions of interest might be evaluated (or re-evaluated) based on clinical presentation and/or previous sonographic findings. Focused techniques at point of care are well-suited to rapid patient screening and/or repeated assessment. More detailed or advanced imaging might be necessary to better characterise ambiguous or inconclusive results following point of care (practitioner) assessment.

Descriptions of sonographic techniques for, and findings from, abdominal assessment of the horse began appearing in equine veterinary publications, conferences and textbooks in the 1980’s and 1990’s [2,20,21,22,23,24,25]. Sonographic evaluation of the abdomen can be completed by specialist veterinarians in a referral capacity or patient-side in a hospital, clinic, stable or ambulatory setting (Figure 2). Published techniques range from focused assessment through discrete sonographic windows [4,8,9] to more systematic and methodical evaluation of both sides of the abdomen (Figure 2). Sonographic penetration is greatest at low frequencies and, although high-frequency linear (transrectal) transducers may permit recognition of superficial abdominal structures [26,27], curved linear or convex transducers of 2–3 MHz are recommended for abdominal assessment of adult horses [22,23]. Even at this low frequency, penetration is typically limited to 20–25 cm, meaning that a substantive mass of axial abdominal viscera is not amenable to sonographic assessment, a constraint that limits the clinical evaluation of horses. In addition to the size and depth of the abdomen, inherent limitations to sonographic assessment relate also to the partial enclosure of the abdomen by ribs and the degree of gas distension often present in a horse with colic [23]. Image quality is optimized when there is good contact between the transducer and skin. This can be facilitated by clipping the hair, wetting the coat with water or ethanol (which also removes oily secretions from the skin and hair) and/or the use of sonographic coupling gel, as noted in the methodology.

**Figure 3:**
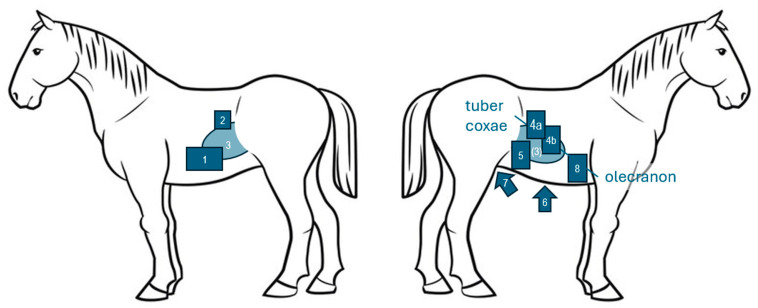
Approximate location of sonographic windows routinely used by the authors: 1—gastric window; 2—nephrosplenic window; 3—left mid-abdomen, (3)—right mid-abdomen; 4a—nephroduodenal window, 4b—hepatoduodenal window; 5—right caudal abdomen (caecal window); 6—ventral abdomen; 7—right (or left) inguinal window; 8—cranioventral thorax. Windows are adapted from published techniques and described in detail (Table 1, and in Supplementary Material). Probe position and orientation should be manipulated to optimise the image in the region of interest.

After reviewing systematic and focused techniques, and based on our collective experience, the acoustic windows of greatest value in the authors’ hands are presented in Table 1. In addition to the seven locations recommended by Busoni et al. in their often-cited focused technique (fast, localized abdominal sonography of horses, FLASH [4]), the authors routinely include evaluation of the left (or right) inguinal region, as a location where jejunal loops are commonly observed, and to ensure assessment of the inguinal canal in appropriate cases. A brief description of the associated technique and representative images from each location are presented in Table 1, and in greater detail in Supplementary File S2 (https://doi.org/10.26188/31858948, accessed on 27 May 2026). The approximate location of each sonographic window is illustrated in Figure 3. By convention, the authors position the probe such that transverse imaging is used preferentially and dorsal is to the left of the image for lateral abdominal windows. Imaging between ribs may necessitate rotation of the probe to orient the transducer within the intercostal space. Transverse or longitudinal images are obtained for ventral windows. Horses tolerate sonographic examination well, with restraint necessary only to the extent expected of other non-invasive interventions such as auscultation.

**Figure 4:**
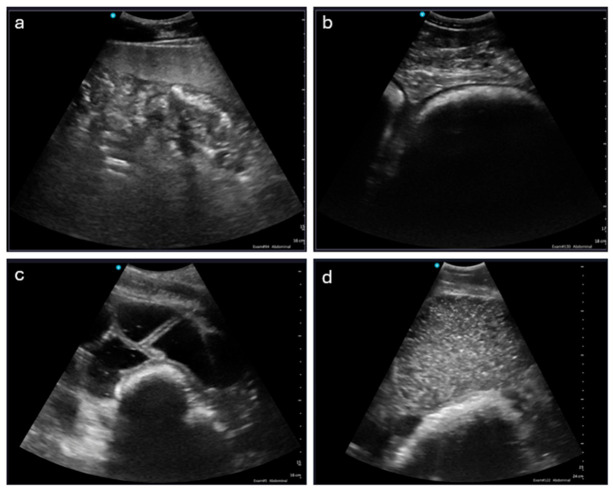
Sonographic appearance of normal intestine. (**a**) Left lower mid-abdomen (ventral part of region 3 in Figure 2); mucus pattern showing collapsed jejunum deep to spleen and containing hyperreflective core of mucus; (**b**) Ventral abdomen showing hyperechoic reflective line typical of normal faecal or gas patterns; only the superficial wall of the colon is visible (note sacculations), with attenuation of deeper content and structures; (**c**) Right inguinal region: fluid pattern in jejunal loops showing intestinal lumen with moderate distension and containing anechoic content; a normal faecal pattern is evident in the colon, deep to jejunal loops; (**d**) right lower mid abdomen (ventral part of region (3) in Figure 2) showing mixed fluid pattern in dorsal colon (top of image) with hyperechoic contents; a normal faecal pattern is evident in the ventral colon (located more deeply in this horse, bottom of image); movement of fluid content is readily apparent during image acquisition (and in cineloops in Supplementary File S2).

Consistent with the findings from human patients [34], small intestinal content in the horse typically has a mucus pattern (collapsed bowel containing only a highly reflective core of mucus with target appearance on cross section) or a fluid pattern (the bowel is filled with fluid and small flecks of content). A gas pattern (only the proximal side of the bowel wall is visible due to beam attenuation by gas) is more common in the large intestine (Figure 4). From a clinical perspective, in the authors’ hands, motility is graded objectively in a manner similar to auscultatory findings (0 = no motility observed, 1= decreased motility, 2 = normal motility and 3 = increased motility [35]). Studies in normal horses demonstrate poor to moderate intra- and inter-rater reliability for measurements of wall thickness [36,37], but suggest that a value of >1mm is sufficient to differentiate measurement variability from true increases in wall thickness [36]. Intra- and inter-observer agreement of motility is moderate, approximating that observed for abdominal auscultation [35].

The duodenum is usually flattened by adjacent viscera and is routinely visualised only during peristalsis. When viewed in transverse section, the duodenum typically has approximately 2 to 5 axial distensions and contractions per minute [38], with lower values reported after withholding feed [2], sedation [35] or transportation [39]. Duodenal contractions are increased for approximately 20 min following nasogastric administration of fluid [40] and fluid content is readily visualised. Motility has been described as ‘normal’ if ≥3 distensions/minute are observed [27] but, in the authors’ experience, abnormal motility has been identified in horses with an increased number of feeble or incomplete contractions, prompting qualitative assessment (increased: contractions of prolonged duration and/or increased amplitude; normal: contractions of expected duration and amplitude; decreased: contractions of reduced duration and/or amplitude; absent: no real contractions observed [35,39]) to more accurately characterise duodenal motility. Aboral movement of ingesta is noted occasionally, and characterization of movement using B-mode or Doppler imaging might improve characterization of duodenal function [3,41,42]. The diameter does not typically exceed 3.4 cm during peristaltic propulsion of ingesta (distension) in healthy horses [2]. During the contraction phase, a central star-shape due to mucosal invagination is often evident, and the measured duodenal diameter is typically ≤1.7 cm [2]. Duodenal wall thickness is typically <3 [43] or 4 mm [1].

Average jejunal loop diameter is usually 2 cm [7,44], and jejunal distension has been described as partial if observed loops have a square or angular profile, and complete when they assume a round shape [45]. Distended jejunal loops may measure 4 to 5 cm. Jejunal motility is typically assessed subjectively, with normal to increased motility recognised by continuous or near continuous movement, including alteration in lumen size and movement of content [35,44]. A star-shape due to mucosal in-folding, similar to that observed during duodenal contraction, has been reported in ex-vivo studies of the jejunum [46]. Jejunal wall thickness should be similar to that reported for the duodenum (≤3 mm [7,36,44]). Wall thickness should be measured from the mucosal surface to the hyperechoic serosal surface or may be measured from lumen to lumen between adjacent bowel segments (and halved). In the authors’ opinion, small intestinal wall thickness is best assessed in moderately distended bowel, as measurements in collapsed bowel segments are likely to be unreliable. Due to its axial location, the ileum is not routinely imaged [1].

The caecum is sacculated and recognised by lateral vessels (Table 1) oriented in a cranioventral–caudodorsal direction and typically located in the right flank immediately caudal to the ribs. The base of the caecum is often gas-filled, precluding assessment of content or medial wall, and is located dorsally, adjacent the right kidney (right 16–17th ICS). The caecum curves cranially, ventrally and axially along the right body wall, allowing visualization of the lateral caecal wall and fluid content. In the normal horse, the right and left ventral colons are recognised by their ventral location and sacculations, with sonographic silhouettes similar to that of the caecum. Mesenteric vessels of the right ventral colon are located medially, and hence are visualised laterally only when the colon is malpositioned [30,31,32]. The right dorsal colon is recognised as a curved structure adjacent to the liver on the right side, and the left dorsal colon is variably visible on the left, typically deep to the spleen [1]; the dorsal colon is non-sacculated. Content of the large colon may be fluid, gas or solid ingesta. Sand may be recognised ventrally by loss of sacculations (flattening) and hyperechoic content with acoustic shadowing [5,47]. Large intestinal contractions (caecum and ventral colons) are recognised by movement of the intestine away from the abdominal wall [35,48] and by movement of fluid intestinal content. ‘Normal’ motility has been defined as ≥2 contractions/minute [48], and by the authors as movement for ≥50% of the observation period [35]. Motility of the left ventral colon is less than that of the caecum and the cranial ventral colon (region of sternal flexure) [49]. The far wall of the large colon can seldom be imaged, precluding routine measurement of lumen size, but distension of the ventral colon can be recognised by the loss of ventral sacculations [45]. Caecal and large colon wall thickness in clinically normal horses and ponies is 2 to 4 mm [43,50]. Severe large colon thickening (>9 mm) has been associated with displacements, strangulating and inflammatory lesions [5]. The small colon is characterized by its diameter and tighter sacculations [1] and is often recognised as short, sharply curving hyperechoic lines in the left paralumbar fossa. Whilst small colon content may be sonographically characterized (fluid, gas or solid ingesta), motility is difficult to assess. The transverse colon is not usually identifiable via transabdominal ultrasonography [1].

### 3.2. What Is the Value of Ultrasound for the Assessment of the Equine Abdomen?

Despite some initial circumspection on the value of the technique [26], ultrasonography is highly recommended in the routine examination of the equine abdomen [5]. Sonographic assessment of the equine abdomen may facilitate recognition of gastric distension, small intestinal distension and ileus, thickening of the large intestine, right dorsal colitis, intraabdominal masses and neoplasia, intraabdominal abscesses, intussusceptions, intraluminal ascarids, inguinal herniation, impactions, entrapment of the large colon in the nephrosplenic space, liver lobe torsions, cholelithiasis, diaphragmatic hernias, displacement of the colon, peritonitis, intraabdominal haemorrhage, and intestinal perforation [23,27]. Although there is little objective information relating sonographic findings and specific abdominal disease presentations, significant associations have been reported between sonographic determination of distended and non-motile intestinal loops (with and without increased free peritoneal fluid) and strangulating small intestinal lesions, a failure to visualize the left kidney with nephrosplenic entrapment (NSE), and thickened large colon with strangulating volvulus [45]. In horses with colic, transcutaneous sonographic evaluation has been reported as more likely to identify large colon volvulus and small intestinal obstruction than rectal palpation, but rectal palpation is more useful for colon displacements, impactions and diseases of the caecum [6,51].

Gastric distension is readily appreciated on sonographic evaluation of the left cranial abdomen, with the stomach normally imaged in the 9th to 13th intercostal spaces in the mid to lower abdomen. Gastric fluid accumulation (horizontal air-fluid interface presenting as a vertical line due to probe orientation) and partitioning of content may be noted (Table 1). The sonographic footprint of the stomach (i.e., the number of intercostal spaces and, to a lesser degree, the maximum height of observed fluid levels) has been correlated with the volume of reflux in horses presenting with gastric distension secondary to intestinal dysfunctional or obstructive conditions [52]. Nasogastric intubation and the administration of fluid increases cranial to caudal and dorsal to ventral gastric dimensions, and fluid content can be seen in the gastric lumen [29,50]. Gastric impaction may be more difficult to recognize sonographically and should be confirmed endoscopically. Serial sonographic assessment may be useful to monitor resolution of impaction.

Small intestinal obstructive lesions are identified by luminal distension, abnormal motility and mural thickening [5,7]. Although most reliably imaged in the inguinal region, distended small intestine may be evident in any part of the abdomen, with the location and extent of abnormal findings largely dependent on the location and type of lesion. In rare instances, obstructive lesions such as intussusception, lipoma, ascarid impaction or hernia may be visualized directly. Strangulating lesions present as mural thickening and oedema with or without vascular distension due to venous occlusion; motility may be decreased, although this is a common non-specific finding in the absence of mural thickening. In addition to strangulation, mural thickening of the small intestine may be associated with infarctive lesions, inflammatory conditions [53] or *Lawsonia* infection.

Nephrosplenic entrapment (NSE, left dorsal displacement of the large colon) can be recognized when there is gas distended large colon obscuring the dorsal aspect of the spleen [5], although it can be difficult to differentiate gas distension of the colon from true displacement. The left kidney may also be obscured and the spleen displaced ventrally although, in the authors’ experience, these findings are less specific. Right dorsal displacement is recognized sonographically when mesenteric colon vessels are visualized directly adjacent to the right body wall, typically coursing horizontally between the 12th and 17th ICS at approximately the level of the costochondral junctions [30,31], although the location of these vessels and the number of intercostal spaces may vary [32]. Anatomically, these vessels are located on the medial aspect of the colon and hence are usually obscured by colon content. They must be differentiated from the lateral caecal vessels [5] which, in the normal horse, run cranioventrally in the right flank caudal to the costal arch [32]. Vascular distension, and mural thickening, are variably present in cases of displacement (with or without torsion) and are likely dependent on the degree of vascular occlusion. In addition to torsions, generalized thickening of the colon wall may be associated with (typhylo)colitis, cyathostomiasis, inflammatory or infiltrative conditions (IBD), whereas focal thickening is more commonly associated with infarctive lesions or non-steroidal toxicity. In the authors’ experience, fluid content in the large and small colons, with or without mural thickening, may be appreciated prior to the development of overt diarrhoea in horses with colitis. Sonographic findings are necessarily interpreted with consideration of history, clinical progression, physical examination and other diagnostic findings.

Ultrasound examination is uniquely suited to subjective assessment of the volume and characteristics of peritoneal fluid, allowing recognition of conditions associated with increased fluid accumulation such as hemoperitoneum [54] and peritonitis [23]. Foetal well-being, characteristics of foetal membranes, allantoic and amniotic fluid [37], and post-partum uterine involution may be evaluated and monitored using this approach [55].

### 3.3. What Is Point of Care Ultrasound, and How Is It Used in Human Healthcare Settings?

Point of care ultrasonography (POCUS) in human medicine is defined as advanced diagnostic sonographic imaging, performed and interpreted by the attending physician as a bedside test [56]. The technique is widely used in many disciplines, especially emergency medicine, as a rapid diagnostic tool [57]. Despite the recent incorporation of POCUS approaches into human healthcare settings, there is already evidence of improved assessment and management of internal medicine patients, as well as resourcing benefits associated with fewer diagnostic imaging requests after a POCUS patient assessment [58]. In a prospective observational study, POCUS evaluation yielded clinically significant sonographic findings in more than one-third of patients, with abdominal abnormalities second in clinical relevance only to cardiac findings [59]. In this study, the clinical management of over one-quarter of patients was changed on the basis of sonographic assessment.

Evaluation of the abdomen using POCUS in human health care settings is accurate for the detection of free abdominal fluid and for assessment of liver pathology and splenic enlargement [60] but may not improve diagnostic accuracy in patients presenting for evaluation of acute abdominal pain [61]. Improved clinical outcomes are likely to be derived from increased practitioner familiarity with the technique, and with increased understanding of common pathologies [12,62,63]. The technique promises to improve patient care in austere, resource-limited healthcare settings, including military combat zones, low-resource environments such as the desert or tropics, microgravity, and high altitudes [28], which are perhaps more analogous to veterinary field situations than other well-staffed and well-resourced human healthcare settings. However, there remains a need for additional high-quality studies to guide POCUS training, disseminate use in non-hospital settings, and maximize impact for improved clinical outcomes [64]. To date, direct comparisons between POCUS and specialized diagnostic imaging findings or clinical accuracy are relatively uncommon in the medical literature. Available POCUS technology has been prospectively evaluated in a comparison of nine different hand-held ultrasound devices (Butterfly iQ+, Clarius C3HD3, D5CL Microvue, Philips Lumify, SonoEye Chison, SonoSite iViz, Mindray TE Air, GE Vscan Air, and Youkey Q7) by Merkel at al [65], who reported that the Vscan Air and SonoEye Chison achieved the best ratings for B-scan quality, handling, and software usability.

### 3.4. What Is the Role of POCUS for the Assessment of the Equine Abdomen in Clinical Practice and Research?

The most common use of abdominal sonography in clinical practice is the assessment of the colic patient using specific anatomic windows to screen patients for specific conditions, with more systematic, complete abdominal sonographic assessment reserved for other clinical scenarios [5]. The small size and ready mobility of POCUS equipment greatly facilitate field assessment or repeated evaluation of hospitalized horses without moving them to a central facility, thereby permitting examination without compromise to patient care or hospital biosecurity. As bacterial contamination of ultrasound transducers has been demonstrated in human health care settings [66], placing the transducer in a plastic sleeve (as described above to protect from ethanol damage) and, if necessary, covering the phone or tablet in plastic (we use zip-lock bags) will also facilitate infection control.

In addition to expediting the diagnosis of specific presentations, serial sonographic assessment can be useful for monitoring case progression or resolution [67]. Serial monitoring of duodenal contractions [38] or jejunal content, wall thickness and motility [67] are useful predictors of post-operative reflux and recovery following colic surgery, and prolonged colon wall thickness following surgical correction of large colon volvulus has been associated with the development of multiple organ dysfunction [68]. In the authors’ experience, gastric distension due to fluid accumulation is detected on sonographic assessment with sufficient reliability as to render unnecessary the passage of a nasogastric tube to assess for reflux in the majority of cases, particularly if there are safety concerns related to this intervention, such as a poorly handled horse or biosecurity concerns (for example, in Australia, it would be considered unsafe in some circumstances to pass a nasogastric tube in a patient with uncertain Hendra vaccination status).

Although the authors used only the Vscan Air CL for the purpose of the current study, at the time of publication, hand-held ultrasound devices that are potentially suitable for use in horses are identified in Table 2. The authors have used only the Vscan, Lumify and Butterfly transducers for sonographic assessment of equine patients, and other probes should be evaluated for this purpose prior to purchase. Focused POCUS protocols have recently been published for abdominal assessment of horses [9,10,69]. These techniques are not substantively different to views routinely obtained by the authors (Table 1). In most cases, there is little deterioration in image quality compared to images from larger, hospital-based equipment [15], with much greater ease of examination. Acoustic coupling with ethanol (or isopropyl) saturation of the coat is usually sufficient for adequate image quality, but it should be noted that veterinary use of ethanol to facilitate abdominal ultrasound in the horse has been associated with positive exhaled breath alcohol tests [70]. Clipping may occasionally be necessary for draft breed horses with coarse coats or horses with long winter coats and can be expected to enhance image quality [69], reduce alcohol required or to facilitate repeat evaluation of an area of interest.

Results of abdominal assessment of horses using a POCUS approach have been compared qualitatively with images obtained from larger, hospital-based ultrasound machines only once in this species [15] and, as yet, there are no studies comparing diagnostic accuracy between specialist imaging services and POCUS, or relating POCUS findings to surgical or necropsy outcomes for horses. As the most common use of POCUS in a field setting is targeted examination using specific anatomic locations [5], the adaptation of existing guidelines for focused sonographic assessment [4,8] is likely to be of most value to the practitioner. As is the case in human healthcare settings [14], the rapid development and ready availability of affordable hand-held POCUS equipment mandate further characterization of the diagnostic accuracy, indications for, and training required for sonographic assessment of the equine abdomen [5,9,15,71]. Although the technology lends itself to remote assistance using telehealth applications, a recent equine study has demonstrated that specialist support for veterinarians undertaking abdominal POCUS in horses was not well accepted by users [72], emphasizing the need to promote student and practitioner development of sonographic skills to complete POCUS examinations autonomously.

Sedation is commonly used to facilitate veterinary examination or treatment of horses with colic, and the adverse effects of α2 adrenergic agents on gastrointestinal motility are well recognized [35,73]. Sonographic assessment of motility has been used previously to demonstrate motility changes following administration of these agents [3,35,73], characteristically reducing motilty and associated with the appearance of an increased volume of fluid small intestinal content [35]. Sonographic imaging has also been used to assess changes attributed to the administration of Buscopan (hyoscine) compositum [33,74] and other pharmacological interventions [46,75], anaesthesia for non-abdominal surgery [76], and the effects of feeding [2], gastroscopy [77] and nasogastric administration of fluid [78]. Whilst these changes are unlikely to confound the clinical interpretation of sonographic findings in colic cases, they should be considered in the context of repeat assessment or equivocal findings.

## 4. Discussion

The current study was undertaken to review the use of POCUS for the field or clinical evaluation of the adult horse presenting for evaluation of acute abdomen and to provide a resource for the practicing veterinarian. Based on the available literature and authors’ experience, POCUS techniques using readily available wi-fi sonographic probes are well-suited to extend the clinical assessment of horses with colic and are a viable alternative to protracted systematic ultrasound examination using large, referral-level equipment. Although a small difference in image quality is appreciated in some patients, clinician-directed sonographic examination is advantageous in a critical care setting as it is readily implemented and easily repeated, eliminates the need to move the horse to the equipment, and facilitates infection control. Further, the modest size and price of POCUS equipment mean that the procedure is readily implemented in general and ambulatory practice settings. Sonographic examinations are well tolerated, and readily conducted in the field, stall or emergency room. Whilst POCUS approaches most readily lend themselves to focused examination protocols, the technology also supports more thorough and systematic examination.

Small intestinal and gastric fluid distension, mural thickening, characterization of intestinal or colon content and motility, increased free abdominal fluid, evaluation of the late-term foetus or post-partum assessment of uterine involution are readily appreciated using transcutaneous sonographic assessment, whereas impactions and large colon displacements might be more amenable to rectal examination. Sonographic assessment, therefore, does not replace rectal examination, but may be a safer or more appropriate diagnostic intervention in some circumstances—for example, ambulatory consultations where rectal palpation may be considered unsafe. Similarly, the technique does not preclude passage of a nasogastric tube to check for gastric reflux, but may also decrease reliance on this more invasive procedure. Specific abnormalities, such as vascular changes associated with right dorsal displacement of the colon [32], might be missed using focused examination techniques. Systematic and more comprehensive sonographic evaluation, and other diagnostic procedures, should be considered to characterize the patient with equivocal or discordant POCUS findings.

### Limitations

Bright light conditions are limiting for visualization of sonographic images, and the technique is further limited by equipment and anatomic factors that preclude examination of some abdominal structures. Although sonographic findings may be operator dependent, measures of intra- and interobserver agreement have demonstrated acceptable repeatability [35,36,37], and reliability of the technique is likely to be increased by enhanced practitioner familiarity with the technique. Further studies might assess the diagnostic accuracy and impact of POCUS on patient outcomes [79], evaluate the effectiveness of post-graduate training in the technique [80], and characterize the confidence and competence of practitioner use of POCUS. The need for further studies to more precisely define statistical measures of test performance is widely cited in this context [6,9,15,72], although the extent that such findings would be transferrable between hospital populations, where differences in clinical presentations and hence pre-test probably might vary substantially, might reduce the value of such comparisons [5,6]. Detailed comparison of transducers available for equine abdominal POCUS would be advantageous.

In response to calls in the veterinary [15,72] and human literature [12,14] for increased education of practitioners on effective use of POCUS techniques, the current review was intended to provide the practicing equine veterinarian with a resource to encourage and facilitate sonographic assessment of patients. In combination with the authors’ experiences, a narrative review approach was selected over a more rigorous systematic review in order to provide a concise and user-friendly resource. The images presented, including those provided online (https://doi.org/10.26188/31858948, accessed on 27 May 2026, Supplementary File S2), represent a convenience sample of cases and experiences with POCUS and are not intended as a comprehensive guide to sonographic imaging of the equine abdomen.

## 5. Conclusions

In summary, hand-held, wi-fi ultrasound transducers can readily be used in field and clinical settings to facilitate rapid assessment of the equine acute abdomen, augmenting other available diagnostic procedures. Advantages relate to small size, portability and ease of equipment use, with acceptable image quality readily achieved in most situations. Interpretation of findings is grounded in an understanding of abdominal anatomy and pathologic changes expected of specific clinical conditions relevant to the case being evaluated. The procedure should augment other aspects of the veterinary examination and can extend the practitioner’s capacity to assess the patient on initial presentation and/or to monitor progression or resolution of a condition of interest.

## Figures and Tables

**Table 1:** Sonographic windows used by the authors as part of targeted sonographic assessment of the acute abdomen. Links to representative normal images, and common abnormalities in each site, are provided as thumbnails; larger images, video clips (cineloops) and further information are provided online https://doi.org/10.26188/31858948, accessed on 27 May 2026 (Supplementary File S2). Ventral abdominal images are taken with the probe oriented craniocaudally and with cranial to the left, unless otherwise indicated; all other views are taken as transverse images (angled between ribs if necessary), and with dorsal to the left. Markers to the right of image are cm.

Side	Site	Scanning Procedure	Representative Normal Images	Common Abnormal Findings
Left	1 Gastric window	The normal stomach may occupy 9th to 13th ICS, and is typically situated at approximately the level of the shoulder [1]; after air insufflation, the stomach may be visualised 7th to 16th ICS [28]; scanning is performed dorsal to ventral along each intercostal space; the transducer is oriented transversely (between the ribs) [29], dorsal is to the left of the image. The greater curvature is recognized as a concave structure lying dorsal (to the left of) splenic vessels. The right image shows ingesta-fluid and fluid-air interfaces (arrows, dorsal is to left of image).	Normal stomach 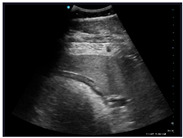	Gastric distension and partitioning of content 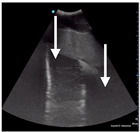
	2 Nephrosplenic window	The transducer is placed between the dorsal and middle thirds of the abdomen at the level of the 17th ICS or immediately caudal to the last rib; considerable individual horse variation may be evident, and the probe should be moved dorsoventrally and/or angled craniocaudally to visualize the left kidney and the epaxial musculature dorsal to this. The nephosplenic space (arrow) may not be visualized clearly in all horses (eg., obscured by lung fields or gas-distended viscus).	Normal nephrosplenic space 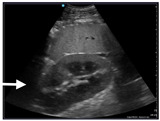	Nephrosplenic entrapment 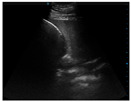
	3 Left (right) middle third of the abdomen	The transducer is moved freely around the middle third of the abdomen, particularly noting any abnormalities in splenic parenchyma, large colon content and mural thickness, or presence of small intestine (jejunum). The spleen is normally visualized adjacent to the left body wall, and jejunal loops may be evident adjacent to this structure (arrows). Distended mesenteric colon vessels are consistent with displacement (+/− torsion) of the colon [5,30,31,32].	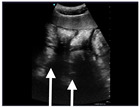	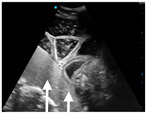
Right	4 Duodenal window	The transducer is placed in the 14–15th ICS in the dorsal part of the middle third (dorsoventral) of the abdomen to visualize the duodenum passing between the liver and right dorsal colon, or moved caudally and dorsally to visualize the duodenum between the cranial pole of the right kidney and the base of the caecum at the 16th and 17th ICS; a line joining the olecranon and the tuber coxa is useful for positioning the transducer in both locations [2], and the transducer is oriented transversely in the intercostal space, parallel to the ribs [33]. Duodenal content, distension and motility are assessed.	4a Nephroduodenal window 4b Hepatoduodenal window	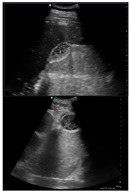
	5 Right caudal abdomen	The transducer is placed between the caudal ribs and moved ventrally past the convergence of ribs to visualize lateral caecal vessels (arrows) coursing cranioventrally caudal to the costal arch; caecal motility and content should be noted. Wall thickening and distension of caecal vessels (right image) are associated with inflammation. The right ventral colon, also sacculated, is located adjacent to the caecum, but mesenteric colon vessels are located medially are cannot normally be visualised.	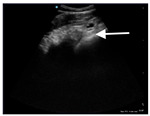	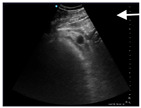
	6 Ventral abdomen	The probe is oriented in a craniocaudal direction, placed on the ventral midline immediately caudal to the xiphoid and moved caudally and/or sagittally to visualize the most gravity dependent abdominal contents. Peritoneal fluid may be evident between sacculations and, if an excessive volume is present, should be assessed for sonographic characteristics (echogenic content consistent with increased cellularity and/or fibrin). Note increased free abdominal fluid and jejunal loops in image right (transducer oriented transversely).	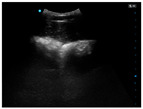	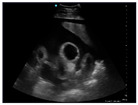
	7 Inguinal region	If visible, the jejunum is most frequently evident in the right or left inguinal region. In compliant horses, both sides can be imaged from the right, but care should be taken as most horses are unfamiliar with application of alcohol or transducer in this location. The presence, motility and content of small intestinal loops should be noted in this window, as should the presence of large colon structures and content.	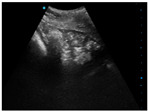	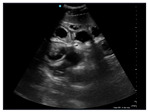
	8 Cranioventral thorax	The probe is placed immediately caudal to the elbow and triceps muscle and angled between ribs to determine if there is abnormal pleural fluid accumulation, pleural thickening or consolidation of the peripheral lung fields. Crude evaluation of cardiac and pericardial structures is also possible, but detailed assessment of thoracic anatomy requires different sonographic approaches. Images right show normal costophrenic angle (left) and pleural effusion (right).	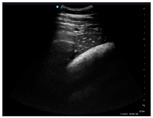	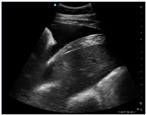

**Table 2:** Handheld ultrasound transducers available to equine practitioners and likely suitable for point of care use; all (except Sono-site iViz) are Android and iOS compatible. The authors have used VScan Air, Lumify and Butterfly IQ probes for evaluation of horses; the suitability of other transducers has not been demonstrated in equine patients to our knowledge.

Company	Model	Frequency & Depth	Wireless/Tethered
GE Healthcare https://www.gehealthcare.com.au/products/ultrasound/handheld-ultrasound (accessed on 27 May 2026)	Vscan Air CL * Vscan Vet (software app)	curved 2–5 MHz max depth 24 cm	wireless
Clarius https://store.clarius.com/products/c3-hd3-handheld-ultrasound-scanner (accessed on 27 May 2026)	C3HD3 multi- purpose scanner	convex 2–6 MHz max depth 40 cm	wireless
ASUS https://www.asus.com/mobile-handhelds/wearable-healthcare/asus-handheld-ultrasound-for-veterinary-care/asus-handheld-ultrasound-lu800-for-veterinary-care/ (accessed on 27 May 2026)	LU800C Vet	convex 2–5 MHz max depth 30 cm	wireless
Butterfly iQ+ Vet https://vet.butterflynetwork.com/ (accessed on 27 May 2026)	iQ3 convex	convex 1–12 MHz max depth 30 cm	tethered
Philips Healthcare https://www.philips.com.au/healthcare/sites/lumify-handheld-ultrasound (accessed on 27 May 2026)	Lumify C5-2	curved 3–5 MHz max depth 30 cm	tethered
Australian Medical Systems https://www.australianmedicalsystems.com.au/vet/wireless-ultrasound---3-in-1-probe (accessed on 27 May 2026)	Wireless Probe 3-in-1 series	convex 3.5–5 MHz max depth 30 cm	wireless
Mindray https://www.mindray.com/au/products/ultrasound/point-of-care/te-air (accessed on 27 May 2026)	TE Air	phased array 2–4 MHz max depth 38 cm	wireless
SonoHealth https://www.sono-health.com/d5cl/ (accessed on 27 May 2026)	D5CL	convex 3.5/5 MHz max depth 30.5 cm	wireless
Chison https://www.chisonsonoeye.com/products/handheld-ultrasound-sonoeye-p5.html (accessed on 27 May 2026)	SonoEye P5	convex 3.5 MHz max depth not stated	tethered
Sono-Site iViz https://www.sonosite.com/au/products/ultrasound-transducers/c60v (accessed on 27 May 2026)	C60v	curved 2–5 MHz 30 cm	tethered (iViz tablet)
Youkey https://www.youkeymedical.com/soloscan (accessed on 27 May 2026)	Q7	curved 2–5 MHz 30 cm	wireless

* This probe was used for images presented in this review.

## Data Availability

The data presented in this study are openly available in Figshare at https://doi.org/10.26188/31858948.

## Supplementary Materials

The following supporting information can be downloaded at: https://www.mdpi.com/article/10.3390/ani16121770/s1, Narrative review methodology is detailed in Supplementary File S1. Supplementary Information (Supplementary File S2) is available for download from https://doi.org/10.26188/31858948 (accessed on 27 May 2026).

## Abbreviations

The following abbreviations are used in this manuscript:

POCUS	point of care ultrasound
POC	point of care
US	ultrasound
FLASH	fast, localized abdominal sonography of horses
MHz	megahertz
ICS	intercostal space
IBD	inflammatory (infiltrative bowel disease)
NSE	nephrosplenic entrapment
mvt	movement
viz	visualised
mm	millimetres
cm	centimetres
