# Supplementary material for: Imaging of the Equine Abdomen Using Point of Care Ultrasound (POCUS)—A Resource for the Equine Practitioner
Source: Animals (Basel). 2026 Jun 8;16(12):1770. doi: 10.3390/ani16121770 (PMC13295562; doi:10.3390/ani16121770)
Supplement: Supplementary file 1 [file animals-16-01770-s001.zip › animals-4253120-supplementary.pdf]

Supplementary File S1

Narrative review methodology

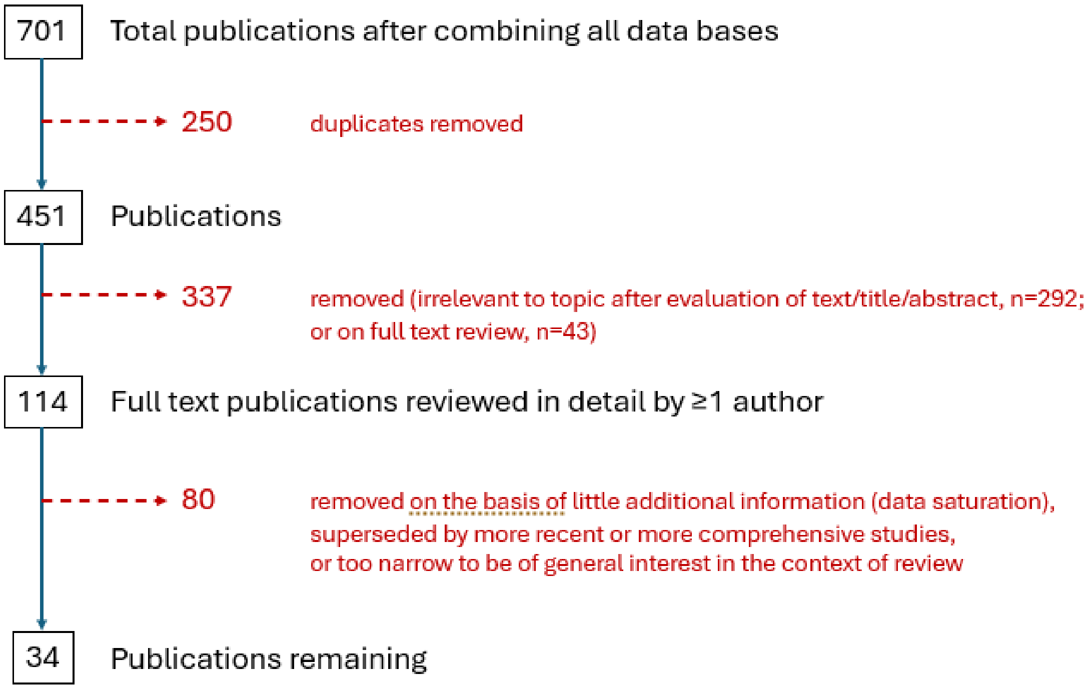

Barton, M.H.(2011) Understanding abdominal ultrasonography in horses: which way is up? Compend Contin Educ Vet 33(9): E1-E8.

Beccati, F., Pepe, M., Gialletti, R., et al. (2011) Is there a statistical correlation between ultrasonographic findings and definitive diagnosis in horses with acute abdominal pain? Equine Vet J Suppl, 2011(39): 98-105.

Bevevino, K.E., Cohen, N. D., Gordon, S. G. and Navas de Solis, C. (2023) Feasibility of a point-of-care ultrasound protocol for cardiorespiratory evaluation of horses in different clinical settings. J Vet Intern Med 37(3): 1223-1232.

Brianceau, P., Chevalier, H., Karas, A., et al. (2002) Intravenous lidocaine and small-intestinal size, abdominal fluid, and outcome after colic surgery in horses. J Vet Intern Med 16(6): 736-41.

Burke, M. and Blikslager, A. (2018) Advances in diagnostics and treatments in horses with acute colic and postoperative ileus. Vet Clin North Am Equine Pract 34(1): 81-96.

Busoni, V., De Busscher, V., Lopez, D., et al. (2011) Evaluation of a protocol for fast localised abdominal sonography of horses (FLASH) admitted for colic. Vet J 188(1): 77-82.

Conwell, R.C., Hillyer, M. H., Mair, T. S., et al. (2010) Haemoperitoneum in horses: a retrospective review of 54 cases. Vet Rec 167(14): 514-8.

Corrie, S., Chapman, K., Schofield, I. and Mair T.S. (2024) Preliminary study to evaluate the use of fast abdominal ultrasonography of horses with colic in first opinion ambulatory practice. Equine Vet Ed 36(11): 571-78.

Cribb, N.C. and Arroyo, L.G. (2018) Techniques and accuracy of abdominal ultrasound in gastrointestinal diseases of horses and foals. Vet Clin North Am Equine Pract 34(1): 25-38.

Cuevas-Ramos, Domenech, G., L. and Prades, M. (2019) Small intestine ultrasound findings on horses following exploratory laparotomy, Can we predict postoperative reflux? Animals 9(12).

Deacon, L.J., Reef, V. B., Leduc, L. and de Solis, C. N. (2021) Pocket-sized ultrasound versus traditional ultrasound images in equine imaging: A pictorial essay. J Equine Vet Sci 104: 103672.

de Solis, C.N. and Coleman, M. (2023) Abdominal sonographic evaluation: In the field, at the hospital, and after Surgery. Vet Clin North Am Equine Pract 39(2): 197-210.

Fischer, A.T., Jr. (1997) Advances in diagnostic techniques for horses with colic. Vet Clin North Am Equine Pract 13(2): 203-19.

Freeman, S. (2002) Ultrasonography of the equine abdomen: techniques and normal findings. In Pract 24(4): 204-211.

Guzmán, J.F.C., Gontijo, A. S., Melgaço, E. S., et al. (2025) Analgesic and gastrointestinal Effects of morphine in equines. Animals 15(4); doi: 10.3390/ani15040571.

Hansen, T., Kendall, A., Finne, R., et al. (2026) Intra-rater and inter-rater reliability of ultrasonographic intestinal wall thickness measurements in healthy horses. Equine Vet J, doi: 10.1002/evj.70147. Online ahead of print.

Haugaard, S.L., McGovern, K. F., Tallon, R., et al. (2023) Ultrasonographic assessment of small intestinal motility following hyoscine butylbromide administration in horses: A pilot study. J Equine Vet Sci 128: 104878.

Hunt, L., Paterson E., Sare H., et al. (2013) The equine gastrosplenic ligament: Anatomy and clinical considerations. Equine Vet Ed 25(1): 15-20.

Klohnen, A., Vachon, A.M. and Fischer, Jr., A.T. (1996) Use of diagnostic ultrasonography in horses with signs of acute abdominal pain. J Am Vet Med Assoc 209(9): 1597-601.

Laus, F., Fratini, M., Paggi, E., et al. (2017) Effects of single-dose prucalopride on intestinal hypomotility in horses: Preliminary observations. Sci Rep 7: 41526.

Leduc, L., Underwood, C., Stefanovski, D., et al.(2024) Evaluation of remote assistance for point-of-care ultrasonography in a large animal hospital: a controlled randomized trial. J Am Vet Med Assoc 262(5): 680-684.

le Jeune, S. and Whitcomb, M.B. (2014) Ultrasound of the equine acute abdomen. Vet Clin North Am Equine Pract 30(2): 353-81.

Lesca, H., Fairburn, A., Fitzharris, L.E. and Mair, T. S. (2023) Ultrasonographic identification of mesenteric lipomatosis in a Shetland mare with recurrent colic episodes. Equine Vet Ed 35(3): p. e186-e192.

Manso-Díaz, G., Bolt, D.M. and López-Sanromán, J. (2020) Ultrasonographic visualisation of the mesenteric vasculature in horses with large colon colic. Vet Rec 186(15): 491-6.

Mitchell, C.F., Malone, E. D., Sage, A. M. and Niksich, K. (2005) Evaluation of gastrointestinal activity patterns in healthy horses using B mode and Doppler ultrasonography. Can Vet J 46(2): 134-40.

Ness, S.L., Bain, F. T., Zantingh, A. J., et al. (2012) Ultrasonographic visualization of colonic mesenteric vasculature as an indicator of large colon right dorsal displacement or 180° volvulus (or both) in horses. Can Vet J 53(4): 378-82.

Norman, T.E., (2024) Maximising the use of point-of-care ultrasonography of the adult equine abdomen and thorax. Equine Vet Ed 36(11): 597-602.

Normandeau, J. (2022) Non-surgical correction of nephrosplenic entrapment and colitis in a Quarter Horse. Can Vet J 63(12): 1255-1257.

Paulussen, E., Broux, B., van Bergen, T., et al. (2018) Caecal intussusception in the horse: Ultrasonographic findings and survival to hospital discharge of 60 cases (2009–2013). Equine Vet Ed 30(5): 241-246.

Santschi, E.M., D.E. Slone, Jr., and Frank 2nd , W.M. (1993) Use of ultrasound in horses for diagnosis of left dorsal displacement of the large colon and monitoring its nonsurgical correction. Vet Surg 22(4): 281-4.

Scharner, D., Rötting, A., Gerlach, K., et al. (2002) Ultrasonography of the abdomen in the horse with colic. Clinical Techniques in Equine Practice 1(3): 118-124.

Tharwat, M. and Al-Sobayil, F. (2025) Equine colic: A comprehensive overview of the sonographic evaluation, diagnostic criteria, and management of different categories. Open Vet J 15(3): 1116-1139.

Vitale, V., Nocera, I., van Galen, G., et al. (2023) Breath alcohol test results in equine veterinarians after performing an abdominal ultrasound with ethanol. Vet Sci 10(3): 222.

Williams, S., Cooper, J. and Freeman, S. (2014) Evaluation of normal findings using a detailed and focused technique for transcutaneous abdominal ultrasonography in the horse. BMC Vet Res 10 (Suppl 1): S5 (http://www.biomedcentral.com/1746-6148/10/S1/S5).
